# Development of a Low-Cost Portable Electronic Nose for Cigarette Brands Identification

**DOI:** 10.3390/s20154239

**Published:** 2020-07-30

**Authors:** Zhiyuan Wu, Hanying Zhang, Wentao Sun, Ning Lu, Meng Yan, Yi Wu, Zhongqiu Hua, Shurui Fan

**Affiliations:** Tianjin Key Laboratory of Electronic Materials and Devices, School of Electronics and Information Engineering, Hebei University of Technology, Tianjin 300401, China; 173817@stu.hebut.edu.cn (Z.W.); 173712@stu.hebut.edu.cn (H.Z.); 184156@stu.hebut.edu.cn (W.S.); 201831903031@stu.hebut.edu.cn (N.L.); ymg08953@163.com (M.Y.); wuyi@hebut.edu.cn (Y.W.); zhongqiuhua@hebut.edu.cn (Z.H.)

**Keywords:** electronic nose, gas sensors, cigarette brands identification, metal oxide (MOX) sensors, sensor array

## Abstract

In China, the government and the cigarette industry yearly lose millions in sales and tax revenue because of imitation cigarettes. Usually, visual observation is not enough to identify counterfeiting. An auxiliary analytical method is needed for cigarette brands identification. To this end, we developed a portable, low-cost electronic nose (e-nose) system for brand recognition of cigarettes. A gas sampling device was designed to reduce the influence caused by humidity fluctuation and the volatile organic compounds (VOCs) in the environment. To ensure the uniformity of airflow distribution, the structure of the sensing chamber was optimized by computational fluid dynamics (CFD) simulations. The e-nose system is compact, portable, and lightweight with only 15 cm in side length and the cost of the whole device is less than $100. Results from the machine learning algorithm showed that there were significant differences between 5 kinds of cigarettes we tested. Random Forest (RF) has the best performance with accuracy of 91.67% and K Nearest Neighbor (KNN) has the accuracy of 86.98%, which indicated that the e-nose was able to discriminate samples. We believe this portable, cheap, reliable e-nose system could be used as an auxiliary screen technique for counterfeit cigarettes.

## 1. Introduction

Gas sensors based on semiconducting metal oxides (MOX) have been successfully used in the detection of various gases [1,2]. The most common MOX gas sensors have a sensitive layer made of tin oxide (SnO_2_) [3], tungsten oxide (WO_3_) [4], or zinc oxide (ZnO) [5]. Since its invention in 1982 [6], the e-nose has been widely used to detect odors. An e-nose system usually consists of: a sensor array including multiple sensors that react in some repeatable way when exposed to volatile substances released by analytes, a data acquisition (DAQ) system for measuring and collecting the responses of sensor array with a computer program which analysis results. This powerful tool has assisted many fields in food analysis [7,8], such as beer quality inspection [9,10], quality level identification of tea [11], characterization of juices [12], and differentiation of aromatic flowers [13]. Additionally, e-nose can be applied to the monitoring of air quality [14,15,16,17,18,19] of gases emitted by the soil [20,21].

Because of widespread imitation cigarettes, the Chinese government and the cigarette industry suffering a multi-million dollar loss in sales and tax revenue annually [22,23], the same problem has occurred in other countries, which indicated it had become a global problem. Emissions of odor from cigarettes contain a great number of volatile organic chemical compounds [24], which make it difficult to distinguish between different brands of cigarettes by the human nose. Additionally, it is challenging to differentiate counterfeit cigarettes only by visual observation. The e-nose can be used to distinguish the different aroma molecules emitted from various cigarettes, indicate that one possible solution to this problem is to develop a suitable e-nose system for cigarette brands identification. Cigarette brand and tobacco analyses using the e-nose system also have been reported in the literature [25,26,27,28]. Cigarettes at different prices have characteristics that distinguish them from each other, and one of the most important is their odor. However, the most performant apparatus for odor detection is cumbersome, expensive, and not entirely dedicated to mobile systems. Most of the researches related to embedded noses for odor detection aims to develop efficient algorithms and newer sensors, but the cost and portability of the device are rarely considered.

**Figure 1:**
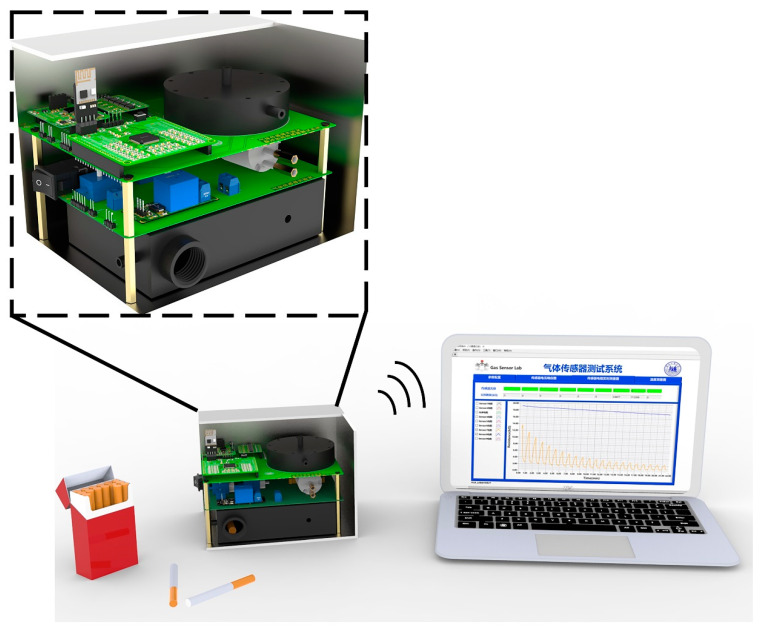
Low-cost portable e-nose system for cigarette brand identification (right side and front opened).

The main purpose of this research is to build a cost-saving and easy to carry e-nose for cigarette brands to discriminate, which can be easily used by cigarette factories, customers, and border agents. In the present case, the setup of e-nose is used to screen counterfeit cigarette. The suspect cigarette is then be checked for Gas Chromatography (GC) and Fourier Transform Infrared Spectrometer (FTIR), which has enough legal validity. A self-developed system (Figure 1) is proposed to identify five different cigarettes. A 16-bit DAQ was designed to be integrated with the sensor array, and the structure of sensing chamber was evaluated by computational fluid dynamics (CFD). Besides, a gas sampling device is proposed to reduce the influence caused by humidity fluctuation and the volatile organic compounds (VOCs) such as ethanol vapor and CO, which is present in the environment due to the daily solvent and emission of mobile. Random Forest and K Nearest Neighbor were employed to analyze the collected data. Considering that the size and cost have a great significant impact on the practicability of the whole system, we reduced the size of our e-nose to a cube with a side length of 15 cm and a cost of only $98.1 (All the costs listed in the Supporting Information Table S1).

## 2. Materials and Methods

The selection of the sensors for the sensor matrix is the first stage in our e-nose construction. Since MOX sensors have drift phenomenon, they need to be replaced frequently. So, the consistency between the different batches of sensors needs to be considered. At the same time, in order to improve the utility of the instrument, the selected sensors must be affordable and can be commercially available. The commercial sensors we selected were characterized by small differences between batches, low cost, and easy availability. Four common sensors (TGS-2620, TGS-2600, TGS-2602, and TGS-2611, Figaro Engineering Inc., shown in Table 1) were obtained from local supplier in Tianjin, China. More specific parameter information can be referred to the website https://www.figaro.co.jp/.

**Figure 2:**
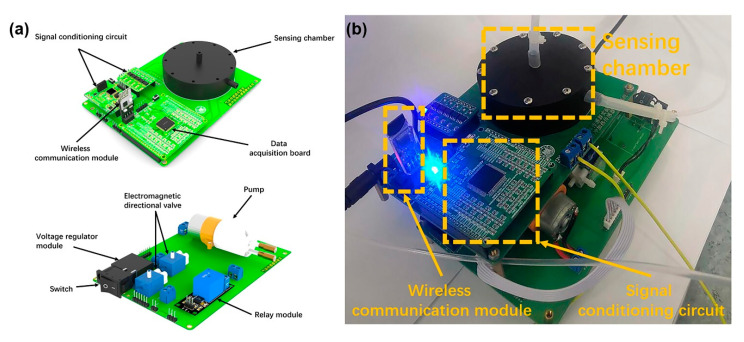
Images are showing (**a**) Sensing chamber with the data acquisition (DAQ) board. (**b**) A physical view of the DAQ system.

The array is embedded into a sensing chamber, which has been design for a good distribution of gas pressure and flow. A DAQ board (Figure 2a) was designed based on A/D convert AD7616BSTZ and MCU chip STM32F103C8T6. The DAQ has a resolution of 16 bit while the input impedance is up to 1 GΩ. Analog signals from sensors were converted to digital signals employing the DAQ board.

The sensing chamber was fabricated by 3D printing using nylon material and HP Jet Fusion 4200 3D printing machine with a resolution of 200 μm. Apart from the sensing chamber, the major components of the e-nose system shown in Figure 2a are pump, electro-magnetic directional valve, sample chamber, and VOC absorbers. A stable and constant gas flow is extremely important for the sensor reading, which is significantly fluctuated with flow rate of air or sample odors. A stable sensor reading is the base of pattern recognition. However, if one considers the low cost and portable e-nose, the cost of mass flow meters (at least two, one for air and the other for sample odors) could be too high to accept. The pump speed is adjusted in real time by the position-type PID algorithm running on the MCU, which ensures the consistency of the pump speed during each measurement. The long-term (five hours) stability of flow rate on sensor readings were also evaluated (Transient response of four gas sensors in five hours is shown in Figure S1 of Supplementary Materials). The gas flow rate of our pump was set to 2 L/min. Two electro-magnetic directional valves were used to switch between two gas paths (acquisition gas path and cleaning gas path).

**Figure 3:**
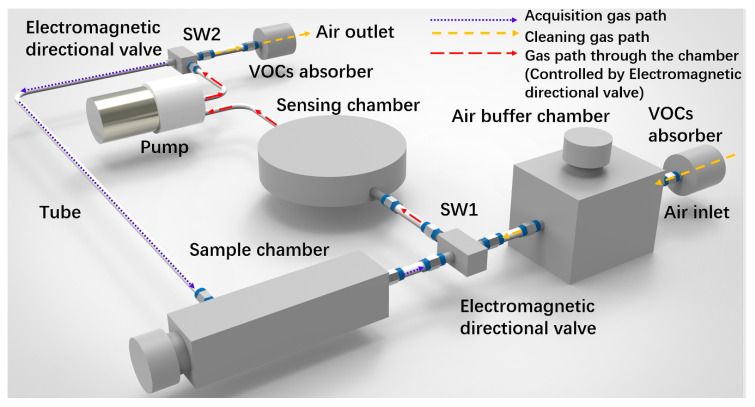
Schematic view of the portable cigarette odor measuring system.

Considering the working environment, interfering gases such as VOCs could be present. For example, ethanol and formaldehyde, which is standard solvent in daily life, could lead to a significant response for MOX sensors. Thus, two absorption devices were installed at the inlet and outlet of the cleaning gas path to absorb VOCs that may have a fluctuation in the sensor base line to reduce the influence of VOCs in the environment (Figure 3). As humidity fluctuations are common but will have a great impact on the MOX sensor, a buffer chamber was designed (shown in Figure 3). When air passes through the VOCs absorber, it enters the buffer chamber. The volume of the buffer chamber is 500 cm^3^, which is enough to reduce the influence caused by humidity change. Different gas passages (acquisition gas path and cleaning gas path) were switched by two electromagnetic directional valves. Controlled by MCU, only one gas path could flow through the sensing chamber at the same time (red line in Figure 3). For each test, odor from the cigarette passes through the chamber along the acquisition gas path (blue line in Figure 3). Electromagnetic directional valves changed the gas path to the cleaning gas path (yellow line in Figure 3). Interfering gases in the environment were eliminated when air passes through the VOCs absorber installed at the inlet.

The main objective of the experiment is to simulate the process of identifying the cigarette brands in a real working environment out of the lab without high purity gas cylinders. To evaluate the validity of the absorbing filter, interference gas ethanol vapor, which is a typical VOCs was flowed into the filter with a high concentration of up to 300 ppm. In minutes 0 to 2 and 4 to 6, VOCs absorber was installed at the inlet of the cleaning gas path. As a comparison, VOCs absorber was removed in a period of 2 to 4 and 6 to 9 min.

**Figure 4:**
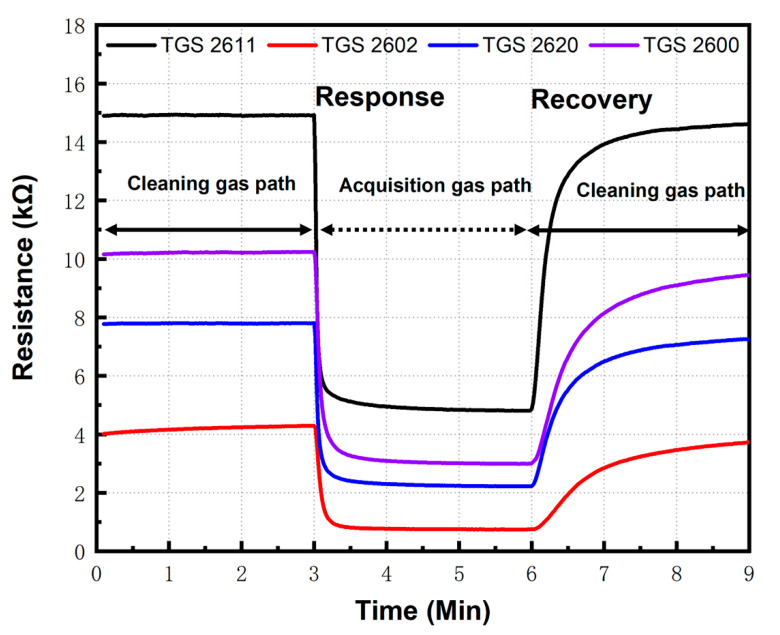
Process of each sampling: electromagnetic directional valve change gas path at 3 and 6 min.

For the identification of cigarettes brands, five cigarettes samples (Table 2) were obtained from local suppliers, which could be divided into two categories. The brands from different producers recognize the first one. The other group is from the same manufacture; however, their price varies with the amount of nicotine. Every measurement, two cigarettes from same brands were placed in the sample chamber. The cigarettes were not lit and gas analysis was done on the odor emitted from unlit cigarettes. Figure 4 shows the process of sampling each time. The measurement cycle consisted of 3 min baseline purge (cleaning gas path, Phase I), 3 min sample draw-in (acquisition gas path, Phase II), and 3 min baseline recovery (cleaning gas path, Phase III). Firstly, air pass through the sensing chamber along the cleaning gas path to obtain a purge baseline. After 3 min, two electromagnetic valves changed the gas path to acquisition gas path. Then, after the period of sample draw-in (3 min), two electromagnetic valves changed the gas path to cleaning gas path again. Air in the air buffer chamber flowed through the sensing chamber along the cleaning gas path and carried the odor out of the sensing chamber.

480 samples were collected in total. The validation is conducted by two common machine learning method: K-Nearest Neighbor (KNN) and Random Forest (RF) classifier analysis.

The KNN algorithm is an effective, easy to understand and non-parametric classification method [29]. By giving a prediction target, the algorithm calculates the distance or similarity between the prediction samples and others, then selects the first K samples that are closest to each other and use these samples to vote for decisions [30]. Random forest is an ensemble learning technique can deal with high-dimensional data without feature extraction. Usually, ensemble learning produces a series of individual weak learners and uses certain strategies to combine them. All results of the weak classifiers would be voted to obtain the final result.

## 3. Results and Discussion

### 3.1. Influence of Chamber Structure on Airflow Distribution

The gas distribution in the chamber was greatly affected by the structure of the chamber. When analyzing the sensing chamber, the first step is to get an overview of the turbulent flow field. The results from the turbulent-flow simulation can then be used for further analyses whether the structure is suitable. In this case, the k-ε turbulence model is used, as it is often done in industrial applications, much because it is both relatively robust and computationally inexpensive compared to more advanced turbulence models. The k-ε model makes use of wall functions to describe the flow close to walls instead of resolving the very steep gradients there.

**Figure 5:**
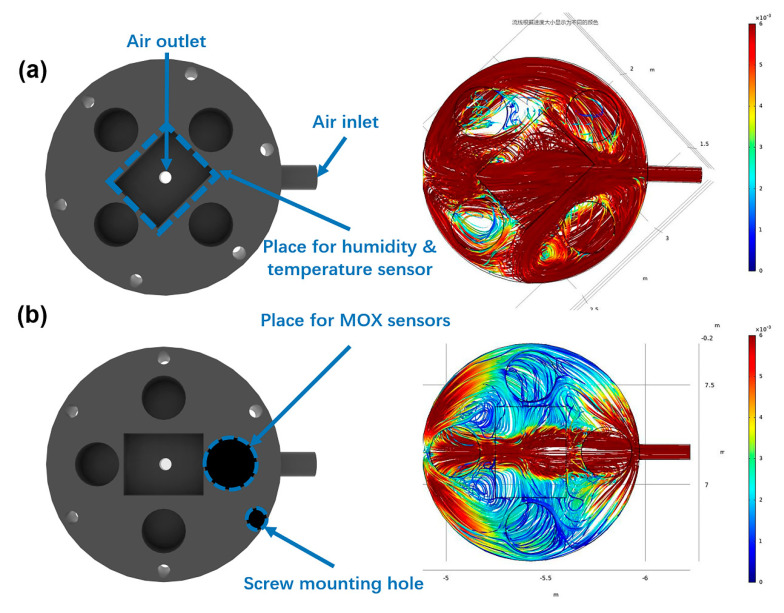
The computational fluid dynamics (CFD) simulation results in different chamber structures. (**a**) Sensing chamber with uniform distribution of internal airflow. (**b**) Sensing chamber with an uneven distribution of internal air flow

The k-ω turbulence model is used to calculate the distribution of air in the chamber with the inlet flow velocity of 1 m/s. Usually, the gas distribution in the chamber with a casual-designed structure is not uniform. This leads to a different sensor reading for the same sensor at a different location in the sensor chamber. Consequently, the result of pattern recognition becomes unrepeatable. In order to ensure the uniformity of airflow distribution in the chamber structure where the gas sensor array is embedded, CFD simulations were used to verify the flow distribution in the chambers of different structures. A typical example is that the structure is shown in Figure 5a has better uniformity than that shown in Figure 5b.

### 3.2. The Effect of Removing Other Interfering Gases from the Environment

**Figure 6:**
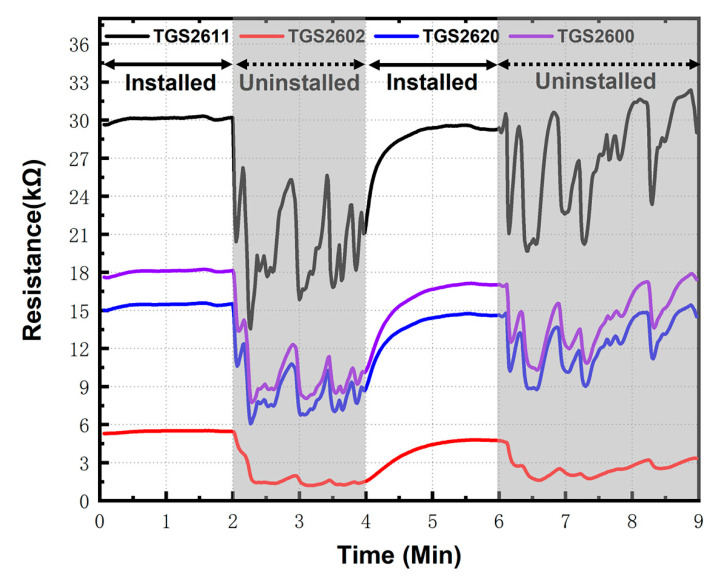
Comparison of volatile organic compounds (VOC) absorption device used in an ethanol environment than not used.

As expected, all sensors respond to ethanol, and the sensor resistances significantly fluctuated with the presence of ethanol in environment as given in Figure 6. This is understandable because ethanol is a typical VOCs, for which MOS sensors are quite sensitive. Indeed, the intensity of response was somewhat different with sensor materials. Thus, it is evident that responses to orders emitted by cigarettes could be shields by VOCs such as ethanol exists in the environment, and the pattern recognition becomes impossible. However, the installation of a carbon filter in the upstream of the sensor array could significantly remove the response of ethanol. The baseline resistance becomes stable as shown in the white background of Figure 6.

One can note that the sensor resistance is quite stable even with a high concentration of ethanol vapor up to 300 ppm. The data were processed to calculate the sensor stable parameter ($S_{0}$) in the ethanol vapor environment. The $S_{0}$ parameter is defined as follows, and it expresses the stability of sensor’s baselines:

(1) $$S_{0}=\frac{\sum_{i=1}^{4}\sigma_{i}^{2}}{4}$$

where $\sigma_{i}^{2}$ denotes the variance of data from four sensors. The result of $S_{0}$ in minutes 0 to 2 (0.02) and 4 to 6 (1.85), which is significantly less than $S_{0}$ in minutes 2 to 4 (4.04) and 6 to 9 (5.55), indicating the carbon filter works very well to remove interference VOCs. This is very important for pattern recognition as the main component of cigarettes are also VOCs.

As synthesized gas can rarely be used to guarantee the stability of the sensor baseline in practical application scenarios, this efficient VOCs absorption device is the key to stabilize the air baseline, which provides a guarantee for high-quality pattern recognition.

### 3.3. Analysis of Sensor Response

**Figure 7:**
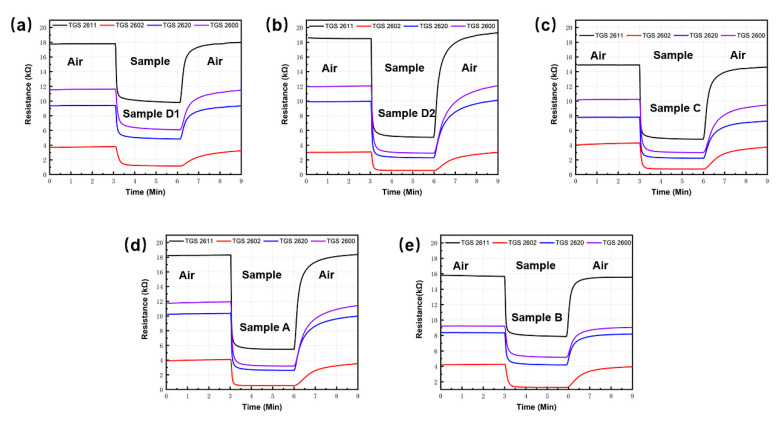
Transient response of gas sensors to different cigarette samples. (**a**) Sample D1; (**b**) Sample D2; (**c**) Sample C; (**d**) Sample A; (**e**) Sample B.

It is found that all sensors give a distinct resistive response to orders of cigarettes, and a set of typical transient response for sensor array was shown in Figure 7. It is not difficult to find that different brands of cigarette have similar sensor responses because the main odor components are alike. However, the detailed response values are different for each kind of cigarette. Sensors show a different response characteristic in the term of responding speed, response intensity, recovering rate. Additionally, we noticed that the average, mean value, and variance of each test were remarkably different, which indicated that the order from different cigarettes is diverse in composition and concentration. This is the condition for pattern recognition.

Four hundred and eighty sets of response data were prepared for pattern recognition of cigarettes. Considering our pattern recognition is operated in a real setting such as factory and cigarette stores. Due to the unexpected variations of temperature, humidity, and air pressure, the baseline of resistance may considerably shift. In order to avoid noise caused by baseline shift, data from the sensors were normalized as follows:

(2) $$D=\frac{D_{0}}{D_{max}}$$

$D_{max}$ indicates the maximum of sensor responses, and $D_{0}$ represents the sensor data before being normalized (A typical Figure of data after normalization is shown in Supporting Information Figure S2). Integral, average value, and variance of each sensor response were calculated as three features for pattern recognition. Three features: the integral (*I*_0_), average (*A*_0_), and variance (*V*_0_), were calculated as follows after the sensor data was normalized:

(3) $$I_{0}=\overset{t}{\underset{t=1}{\sum }}R_{t}$$

(4) $$A_{0}=\frac{I_{0}}{t}$$

(5) $$V_{0}={\left(\frac{\sum_{1}^{t}{\left(R_{t}-A_{0}\right)}^{2}}{t}\right)}^{\frac{1}{2}}$$

where *R_t_* denotes the data after normalized at t second. Each measurement takes *t* second, so the upper and lower limits of the sum is *t*. *I*_0_ is the sum of all the data collected during a single measurement. *A*_0_ and *V*_0_ are the mean value and variance of the data collected for each measurement, respectively. All three features reflect the amplitude of sensor response to some extent.

### 3.4. Pattern Recognition

Two common machine learning methods: K Nearest Neighbors (KNN), Random Forest (RF) classifier, were tested aimed at find the best detection method for data analysis. 480 sample were prepared by the e-nose system for pattern recognition. Then 192 groups (40%) were randomly selected as test set and 288 groups (60%) as training set. Firstly, the original cigarette aroma dataset was separated into training sets and testing sets randomly. Secondly, we use the training sets to build model and test the model with test set. The programs in this paper we developed used Python 3.7 language and the scikit-learn module [31]. The computer operating system was Windows 10 with Intel core i5 processor. The results showed that Random Forest is best suited for this work. As an ensemble learning technique, Random Forest can deal with high-dimensional data without feature extraction, which is a good fit for our dataset.

#### 3.4.1. Random Forest

**Figure 8:**
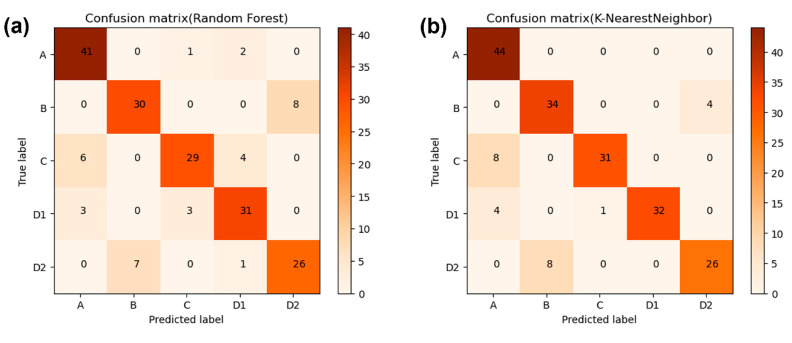
Confusion matrix results of different methods. (**a**) Random Forest classifier. (**b**) K Nearest Neighbors.

As mentioned above, Random Forest is an ensemble learning method, which usually produces a series of individual weak learners. Certain strategies were used to combine those weak learners. The result of Random Forest was obtained by voted from all results of the weak classifiers. The combination of the weak classifiers forms a strong classifier, which can improve the generalization performance of the classification algorithms. The decision tree, as the most used base estimator, is the fundamental unit of Random Forest in our work. The classification and regression tree (CART) were used in this work as the weak classifier. The result of Random Forest classifier in test set is 91.67% and the confusion matrix is shown in Figure 8a. As a result of Sample B, Sample C, and Sample D2, volatilized aroma has similar components, resulting in them being divided into the wrong categories in the verification process.

#### 3.4.2. K Nearest Neighbors

Result of the analysis of KNN method using all the data of all four sensors, are shown in Figure 8b. KNN method is a simple, effective, and non-parametric classification method, also called Reference Sample Plot Method. Then, 192 groups of samples were randomly selected for model validation. Figure 8b shows that K Nearest Neighbors (KNN) has the accuracy of 86.98%. The basic steps of KNN algorithm are as follows: firstly, training samples and test sets from cigarettes’ odor samples were constructed. Secondly, a k value was settled. The distance between the training set samples and the test set is arranged from high to low. Finally, K samples with a small distance are selected as the K nearest neighbors of the test samples to obtain the k classes with the nearest neighbors.

Results from two machine learning methods indicated that MOX sensors network is able to discriminate cigarette brands. Figure 8 shows the Random Forest has the best performance with the accuracy of 91.67%, which means it can give a precise result of cigarette brands identification.

#### 3.4.3. Effectiveness of Sensors and Features 

Each sensor was tested for the contribution to the result of pattern recognition. As mentioned in Section 3.3, we considered that each sensor has three features: integral, average, and variance. Gini coefficient were used to gather the statistics of each feature. All the features can be sorted by their importance during the discriminant task. All branch nodes of each feature have specific Gini coefficient values. The importance of each feature is showed by the decrease in the Gini coefficient value.

**Figure 9:**
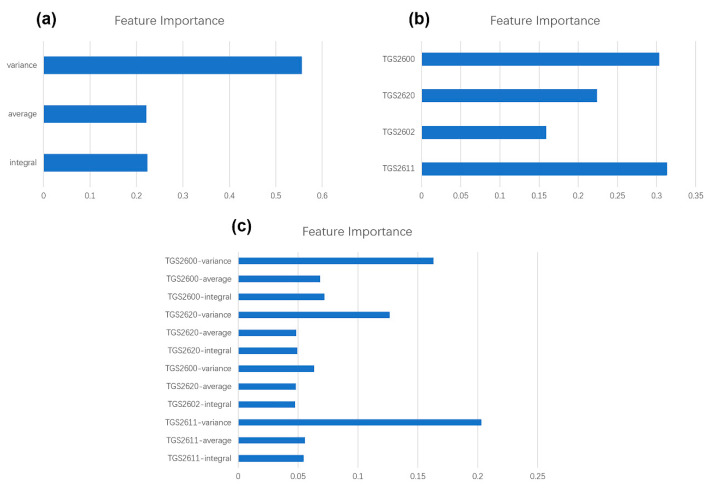
Importance results for different features. (**a**) Feature importance of 3 features we extract (**b**) Feature importance of 4 metal oxide (MOX) sensors in the array. (**c**) Feature importance of all 12 features (4 sensors × 3 features of each sensor).

Figure 9c has displayed the contribution of all 12 features to pattern recognition. We added three features of each sensor to get the contribution of the sensor for the pattern recognition. Figure 9a indicated that variance of sensor response is the most important feature, the integral and average has contributed similar characteristics in pattern recognition. Our work aims to develop a low-cost e-nose system for cigarette brands identification and Figure 9b shows the importance result of each sensor. In our sample database, all sensors showed certain contribution to the result of pattern recognition. Thus, the sensors selected for our e-nose system was proven effective.

## 4. Conclusions

The results presented in our work indicated that using an e-nose system to discriminant different cigarettes is efficient. The e-nose system could not only distinguish different brands of cigarettes but could also distinguish cigarettes of the same brand with different prices, making this method a valuable tool to differentiate genuine cigarettes from counterfeit products. The e-nose we proposed is portable, cheap, and reliable, which are all features essential to field applications carried out by border patrols fighting cigarette smuggling. Additionally, with only a few changes, such as adjusting the combination of sensors, the e-nose can be applied to many other fields such as fruit decay detection and soil environmental monitoring.

We will try to shorten the sample detection time by using pulse modulation to heat the sensor in further study. In the near future, MEMS sensors devices will be used to achieve smaller volume and power consumption. Combine embedded system and IoT platform to make online monitoring and online automatic pattern recognition possible. We will further test other brands, including cigarettes from different countries, to verify our device. At the same time, we will cooperate with relevant departments to carry out field tests to further improve the practicability of the device.

## Figures and Tables

**Table 1:** Characteristics of four sensors selected in our experiment.

Sensor	Target Analyte	Price (USD)
TGS2600	Hydrogen, Carbon mon-oxide	2.8
TGS2602	Ammonia, Hydrogen sulfide	3.1
TGS2620	Alcohol, Solvent vapors	3.4
TGS2611	Methane Natural Gas	3.0

**Table 2:** Cigarette samples used as experimental materials.

Label	Price (USD/Box)
A	2.1
B	2.5
C	5.6
D1	3.1
D2	4.2

## Supplementary Materials

The following are available online at https://www.mdpi.com/1424-8220/20/15/4239/s1, Table S1: The cost of the e-nose, Figure S1. Transient response of four gas sensors in 5 h. Figure S2: Data after normalization.
