# Supplementary material for: Development of a Low-Cost Portable Electronic Nose for Cigarette Brands Identification
Source: Sensors (Basel). 2020 Jul 30;20(15):4239. doi: 10.3390/s20154239 (PMC7435456; doi:10.3390/s20154239)
Supplement: Supplementary file 1 [file sensors-20-04239-s001.pdf]

sensors
MDPI

Table S1. The cost of the e-nose.

Part	Cost (USD)
Printed circuit board	14.7
Sensing chamber	17.1
Electromagnetic valves	1.4
Pump and tube	2.8
Electronic components	21
Sample chamber	18.2
VOCs absorbers	5.6
Device shell	17.3

The long-term stability of flow rate on sensor readings were also evaluated (Figure S1). The sensor resistance was continuously recorded for 5 h, which is thirty times of one single recognition for cigarette (9 minutes). Clearly, the sensor resistance slightly fluctuates with time, which could be caused by the drift of temperatures and humidity of environment.

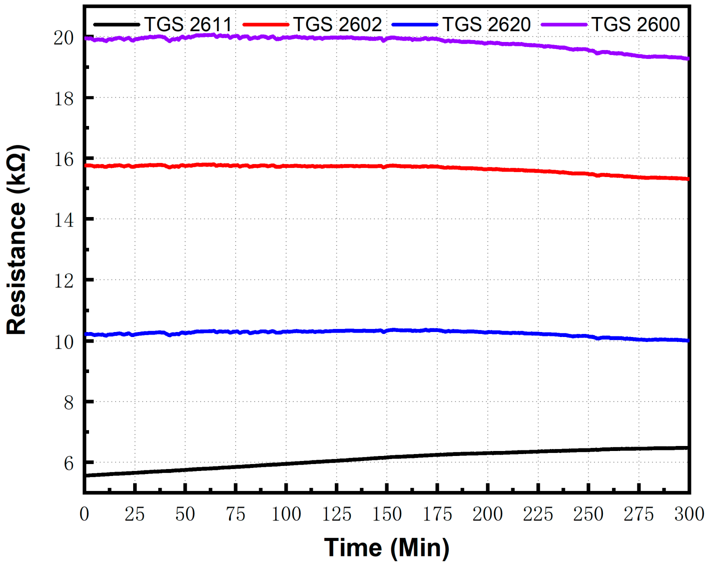

Figure S1. Transient response of four gas sensors in 5 hours.

In order to reduce these problems, the original data from sensors are normalized as follows:

$$D = \frac{D_0}{D_{max}}$$

D_{max} indicates the maximum of sensor responses and D_0 represents the sensor data before normalize. A typical figure of data after normalization is shown in Supporting Information Figure S2.

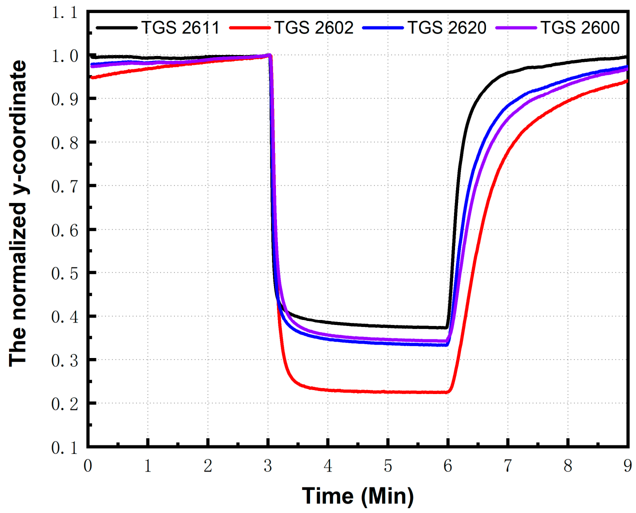

Figure S2. Data after normalization.
